# Extensive sequence duplication in *Arabidopsis* revealed by pseudo-heterozygosity

**DOI:** 10.1186/s13059-023-02875-3

**Published:** 2023-03-09

**Authors:** Benjamin Jaegle, Rahul Pisupati, Luz Mayela Soto-Jiménez, Robin Burns, Fernando A. Rabanal, Magnus Nordborg

**Affiliations:** 1 Gregor Mendel Institute, Austrian Academy of Sciences, Vienna Biocenter, Vienna, Austria; 2 Department of Plant Sciences, University of Cambridge, Cambridge, UK; 3 Max Planck Institute for Developmental Biology, Tübingen, Germany

**Keywords:** Structural variation, Gene duplication, GWAS, SNP calling, Methylation

## Abstract

**Background:**

It is apparent that genomes harbor much structural variation that is largely undetected for technical reasons. Such variation can cause artifacts when short-read sequencing data are mapped to a reference genome. Spurious SNPs may result from mapping of reads to unrecognized duplicated regions. Calling SNP using the raw reads of the 1001 Arabidopsis Genomes Project we identified 3.3 million (44%) heterozygous SNPs. Given that *Arabidopsis thaliana* (*A. thaliana*) is highly selfing, and that extensively heterozygous individuals have been removed, we hypothesize that these SNPs reflected cryptic copy number variation.

**Results:**

The heterozygosity we observe consists of particular SNPs being heterozygous across individuals in a manner that strongly suggests it reflects shared segregating duplications rather than random tracts of residual heterozygosity due to occasional outcrossing. Focusing on such pseudo-heterozygosity in annotated genes, we use genome-wide association to map the position of the duplicates. We identify 2500 putatively duplicated genes and validate them using de novo genome assemblies from six lines. Specific examples included an annotated gene and nearby transposon that transpose together. We also demonstrate that cryptic structural variation produces highly inaccurate estimates of DNA methylation polymorphism.

**Conclusions:**

Our study confirms that most heterozygous SNP calls in *A. thaliana* are artifacts and suggest that great caution is needed when analyzing SNP data from short-read sequencing. The finding that 10% of annotated genes exhibit copy-number variation, and the realization that neither gene- nor transposon-annotation necessarily tells us what is actually mobile in the genome suggests that future analyses based on independently assembled genomes will be very informative.

**Supplementary Information:**

The online version contains supplementary material available at 10.1186/s13059-023-02875-3.

## Background

With the sequencing of genomes becoming routine, it is evident that structural variants (SVs) play a major role in genome variation [1]. There are many kinds of SVs, e.g., indels, inversions, and translocations. Of particular interest from a functional point of view is gene duplication, leading to copy number variation (CNV).

Before Next-Generation Sequencing (NGS) was available, genome-wide detection of CNVs was achieved using DNA microarrays. These methods had severe weaknesses, leading to low resolution and problems detecting novel and rare mutations [2, 3]. With the development of NGS, our ability to detect CNVs increased dramatically, using tools based on split reads, paired-end mapping, sequencing depth, or even de novo assembly [4, 5]. In mammals, many examples of CNVs with a major phenotypic effect have been found [6–8]. One example is the duplication of MWS/MLS, associated with better trichromatic color vision [9].

While early investigation of CNV focused on mammals, several subsequent studies have looked at plant genomes. In *Brassica rapa*, gene CNV has been shown to be involved in morphological variation [10] and an analysis of the poplar “pan-genome” revealed at least 3000 genes affected by CNV [11]. It has also been shown that variable regions in the rice genome are enriched in genes related to defense to biotic stress [12]. More recently, the first chromosome-level assemblies of seven accessions of *A. thaliana* based on long-read sequencing were released [13], demonstrating that a large proportion of the genome is structurally variable. Similar studies have also been carried out in maize [14, 15], tomato [16], rice [17], and soybean [18]. These approaches are likely to provide a more comprehensive picture than short-read sequencing, but are also far more expensive.

In 2016, the 1001 Genomes Consortium released short-read sequencing data and SNP calls for 1135 *A. thaliana* accessions [19]. Several groups have used these data to identify large numbers of structural variants using split reads [20–22]. Here we approach this from a different angle. Our starting point is the startling observation that, when calling SNPs in the 1001 Genomes data set, we identified 3.3 million (44% of total) putatively heterozygous SNPs. In a highly selfing organism, this is obviously highly implausible, and these SNPs were flagged as spurious: presumably, products of cryptic CNV, which can generate “pseudo-SNPs” [23, 24] when sequencing reads from non-identical duplicates are (mis-)mapped to a reference genome that does not contain the duplication. Note that allelic SNP differences are expected to exist *ab initio* in the population, leading to instant pseudo-heterozygosity as soon as the duplicated copy recombines away from its template (as a consequence of outcrossing). In this paper, we return to these putative pseudo-SNPs and show that they are indeed largely due to duplications, the position of which can be precisely mapped using GWAS. Our approach is broadly applicable, and we demonstrate that it can reveal interesting biology.

## Results

### Massive pseudo-heterozygosity in the 1001 Genomes data

**Fig. 1:**
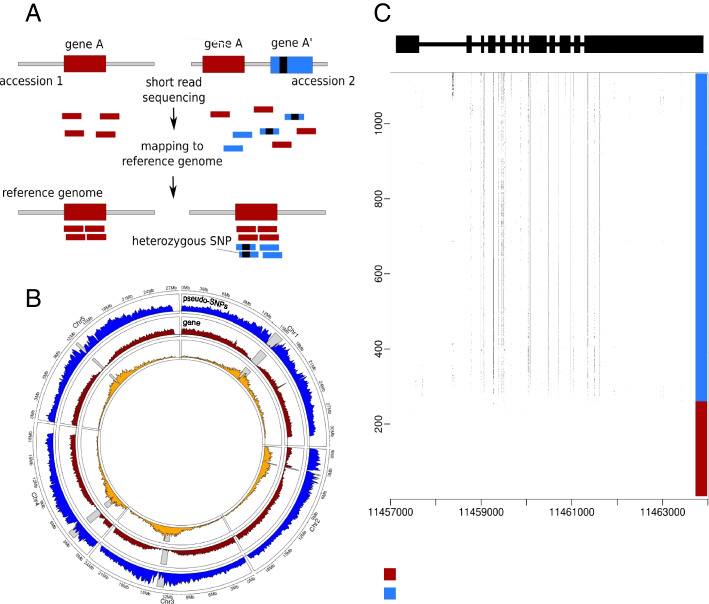
Pseudo-heterozygosity in the 1001 Genomes dataset. **A** Cartoon illustrating how a duplication can generate pseudo-SNPs when mapping to a reference genome that does not contain the duplication. **B** Genomic density of transposons, genes, and shared heterozygous SNPs. Gray bars represent the position of the centromere for each chromosome. **C** The pattern of putative heterozygosity around AT1G31910 for the 1057 accessions. Dots in the plot represent putative heterozygosity

Given that *A. thaliana* is highly selfing, a large fraction (44%) of heterozygous SNPs is inherently implausible. Two other lines of evidence support the conclusion that they are spurious. First, genuine residual heterozygosity would appear as large genomic tracts of heterozygosity in individuals with recent outcrossing in their ancestry. Being simply a random product of recombination and Mendelian segregation, there is no reason two individuals would share tracts unless they are very closely related. The observed pattern is completely the opposite. While a small number of individuals do show signs of recent outcrossing, this is quite rare (as expected given the low rate of outcrossing in this species, and the fact that the sequenced individuals were selected to be completely inbred). Instead, we find that the same SNP is often heterozygous in multiple individuals. Although the population level of heterozygosity at a given SNP is typically low (Additional file 1: Fig. S1), over a million heterozygous SNPs are shared by at least 5 accessions, and a closer look at the pattern of putative heterozygosity usually reveals short tracts of shared heterozygosity that would be vanishingly unlikely under residual heterozygosity, but would be expected if tracts represent shared duplications, and heterozygosity is, in fact, pseudo-heterozygosity due to mis-mapped reads (Fig. 1). Further supporting the notion that pseudo-heterozygous SNPs involve SNPs that already existed in the population before the duplication occurred, both alleles are also present as homozygotes for 97% of the putatively heterozygous SNPs. Analysis of the distribution of the lengths and the number of putatively heterozygous tracts across accessions shows that the vast majority of accessions have a large number of very short tracts (roughly 1 kb) of heterozygosity (Additional file 1: Fig. S2). Longer tracts are rare and not shared between accessions.

Furthermore, the density of shared heterozygous SNPs is considerably higher around the centromeres (Fig. 1), which is again not expected under random residual heterozygosity, but is rather reminiscent of the pattern observed for transposons, where a similar pattern is interpreted as the result of selection removing insertions from euchromatic regions, leading to a build-up of common (shared) transposon insertions near centromere [25]. As we shall see below, it is likely that transposons play an important role in generating cryptic duplications leading to pseudo-heterozygosity (although we emphasize again that the heterozygous SNPs were called taking known repetitive sequences into account).

Despite the evidence for selection against these putative duplications, we found 2570 genes containing 26,647 common pseudo-SNPs (more than 5% [i.e., [26]] pseudo-heterozygous accessions; see (Additional file 1: Fig. S3). Gene-ontology analysis of these genes reveals an enrichment for biological processes involved in response to UV-B, bacteria or fungi (Additional file 1: Fig. S5). In the following sections, we investigate these putatively duplicated genes further.

### Mapping common duplications using genome-wide association

**Fig. 2:**
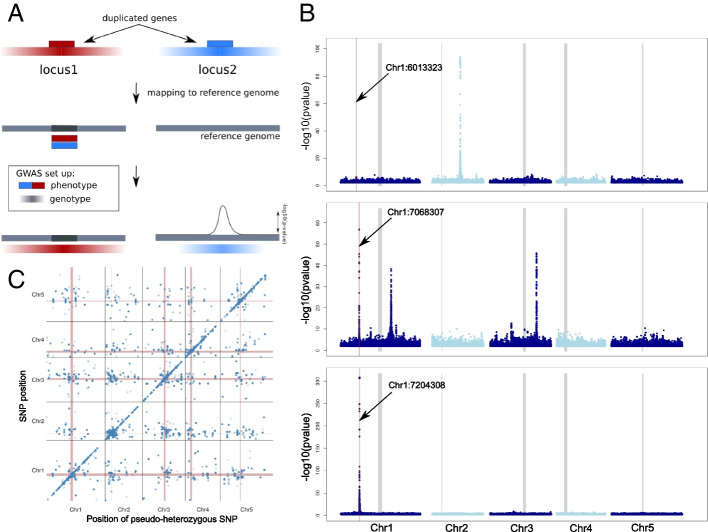
GWAS of putative duplications. **A** Schematic representation of the principle of how GWAS can be used to detect the position of the duplicated genes based on linkage disequilibrium (LD). As phenotype, heterozygosity at the position of interest is coded as 1 (present) or 0 (absent). As a genotype, the SNPs matrix of the 1001 genome dataset was used (with heterozygous SNPs filtered out). Color gradients represent the strength of LD around the two loci. In this example, the reference genome does not contain locus2. **B** GWAS results for three different genes with evidence of duplication, for illustration. The red lines indicate the position of the pseudo-SNP used for each gene/GWAS and the thick gray lines indicate the centromeres. The top plot shows a *trans*-association, the bottom a *cis*-association, and the middle shows a case with both (*cis* plus two *trans*). **C** Summary of all 26,647 GWAS results

If heterozygosity is caused by the presence of cryptic duplications in non-reference genomes, it should be possible to map the latter using GWAS with heterozygosity as a “phenotype” (Imprialou et al 2017). The principle here is that the extra copy that gives rise to pseudo-heterozygosity will be “tagged” by SNPs like any other causal allele (Fig. 2A). We did this for each of the 26,647 SNPs exhibiting shared heterozygosity within genes (Additional file 1: Fig. S3).

Of the 2570 genes that showed evidence of duplication, 2511 contained at least one major association (using a significance threshold of *p* < 10^−20^; see the “Methods” section). For 708 genes, the association was more than 50 kb away from the pseudo-SNP used to define the phenotype, and for 175 it was within 50 kb (the usual extent of GWAS peaks in this species, see also (Additional file 1: Fig. S4). We will refer to these as *trans-* and *cis-*associations, respectively. The majority of genes, 1628, had both *cis-* and *trans-*associations (Fig. 2), suggesting that both the original and the duplicated were tagged by SNPs.

To validate these results, we assembled 6 non-reference genomes de novo using long-read PacBio sequencing. The GWAS results provide predicted locations of the duplications (the putative causes of pseudo-heterozygosity). We identified the homologous region of each non-reference genome, then used BLAST to search for evidence of duplication. For 84% of the 403 genes predicted to have a duplication present in at least one of the six non-reference genomes, evidence of a duplication was found; for 60%, the occurrence perfectly matched the pattern of heterozygosity across the six genomes. For the remaining 16%, no evidence of a duplication was found, which could be due to the stringent criteria we used to search for evidence of duplication (see Methods). The distribution of fragment sizes detected suggests that we capture a mixture of duplicated gene fragments and full genes (Additional file 1: Fig. S6).

### Rare duplications

The GWAS approach has no power to detect rare duplications, which is why we restricted the analysis above to pseudo-heterozygous SNPs seen in five or more individuals. Yet most are rarer: 40% are seen only in a single individual, and 16% are seen in two. As it turns out, many of these appear to be associated with more common duplications. Restricting ourselves to genes only, 11.4% of the singleton pseudo-heterozygous SNPs are found in the 2570 genes already identified using common duplications, a significant excess (*p* = 2.5e−109). For doubletons, the percentage is 11.1% (*p* = 1.9e−139). Whether they are caused by the same duplications or reflect additional ones present at lower frequencies is difficult to say. To confirm duplications, we took the reads generating the singleton and doubleton pseudo-heterozygotes and compared the result of mapping them to the reference genome, and to the appropriate genome (derived from the same inbred line). One predicted consequence of the reads mapping at different locations is that mapping coverage around the pseudo-SNPs will be decreased when mapping to the newly assembled PacBio genomes rather than the reference genome. As expected, a high proportion of the SNPs tested have lower coverage when mapping to the PacBio genomes (Additional file 1: Fig. S7-8). In addition to a decrease in coverage, we were also able to confirm many duplications directly by demonstrating that reads map to multiple locations in their own genome, and that the putative heterozygosity disappeared when we did so. Of doubleton pseudo-heterozygotes, 41.5% are found in regions that are revealed as duplicated simply by mapping to the right genome instead of the reference genome (Additional file 1: Fig. S7-9).

### Local duplications

**Fig. 3:**
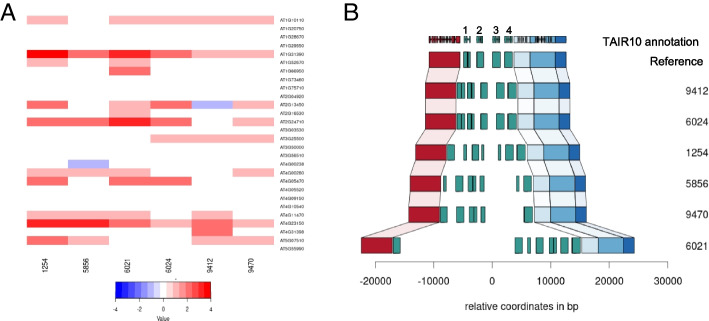
Confirmation of tandem duplications. **A** The distribution of estimated copy number (based on sequencing coverage) across 6 PacBio genomes for 28 genes predicted to be involved in tandem duplications based on the analyses of this paper. **B** The duplication pattern observed in these genomes for the gene AT1G31390, as an example the reference genome contains four copies, shown as numbered green boxes. Other colored boxes denote other genes

If duplications arise via tandem duplications, they will not give rise to pseudo-SNPs until the copies have diverged via mutations. This is in contrast to unlinked copies, which will lead to pseudo-SNPs due to existing allelic variation as soon as recombination has separated copy from the original. We should thus expect the approach taken here to be biased against detecting local duplications. Nonetheless, GWAS revealed 175 genes with evidence only for a *cis* duplication. 28 of these were predicted to be present in at least one of the six new genomes, and 14 could be confirmed to have a local variation of copy number relative to the reference (Fig. 3A).

The local structure of the duplications can be complex. An example is provided by the gene AT1G31390, annotated as a member of MATH/TRAF-domain genes, and which appears to be present in 4 tandem copies in the reference genome, but which is highly variable between accessions, with one of our accessions carrying at least 6 copies (Fig. 3B). However, there are no copies elsewhere in any of the new genomes for this gene (Additional file 1: Fig. S10).

### A transposon-driven duplication

**Fig. 4:**
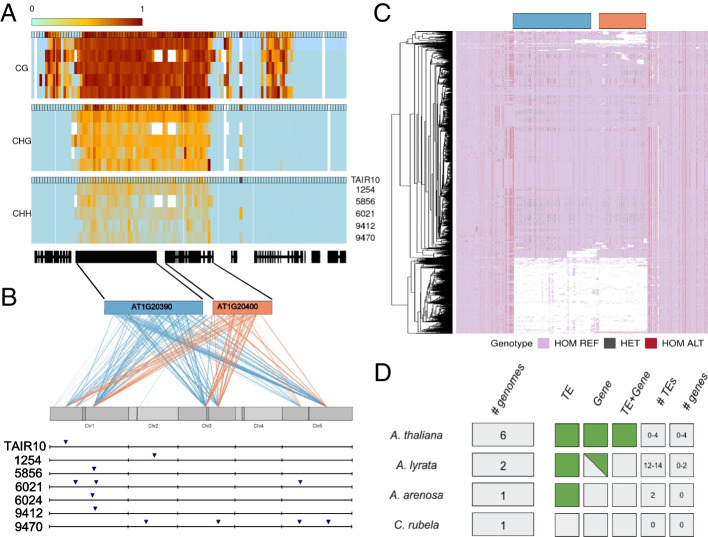
A Gypsy element (AT1G20390) and a gene transpose (AT1G20400) together. **A** Methylation levels on regions containing AT1G20390 and AT1G20400 for 6 accessions, calculated in 200 bp windows after mapping reads to the TAIR10 reference genome (annotation outline in black). **B** GWAS results for the putatively heterozygous SNPs in AT1G20390 and AT1G20400. Each line represents the link between the position of the pseudo-SNP and a GWAS hit position in the genome. The lower part shows the presence of the new transposable element in the 6 PacBio genomes as well as in the reference genome. **C** SNP haplotypes around the AT1G20400 region in the 1001 genomes data. White represents a lack of coverage. **D** Presence of the gene and the transposon in related species

Transposons are thought to play a major role in gene duplications, capturing and moving genes or gene fragments around the genome [27, 28]. While confirming the *trans* duplications in the PacBio genomes, we found a beautiful example of this process. The gene AT1G20400 (annotated, based on sequence similarity, to encode a myosin-heavy chain-like protein) was predicted to have multiple *trans*-duplications. The 944 bp coding region contains 125 putatively heterozygous SNPs with striking haplotype structure characteristic of structural variation (Fig. 4C). We were able to identify the duplication predicted by GWAS in the six new genomes (Fig. 4). Four of the newly assembled genomes have only one copy of the gene, just like the reference genome, but one has 3 copies and one has 4 copies. However, none of the 6 new genomes has a copy in the same place as in the reference genome (Additional file 1: Fig. S11).

In the reference genome, AT1G20400 is closely linked to AT1G20390, which is annotated as a Gypsy element. This element also contains many pseudo-SNPs, and GWAS revealed duplication sites overlapping those for AT1G20400 (Fig. 4B). This suggested that the putative gene and putative Gypsy element transpose together, i.e., that both are misannotated, and that the whole construct is effectively a large transposable element. Further analysis of the PacBio genomes confirmed that AT1G20400 and AT1G20390 were always found together, and we were also able to find conserved Long Terminal Repeat sequences flanking the whole construct, as would be expected for a retrotransposon (Additional file 1: Fig. S12-13). We did not find any evidence for expression of AT1G20400 in RNAseq from seedlings in any of the accessions. Available bisulfite sequencing data [29] showed that the whole region is heavily methylated, as expected for a transposon (Fig. 4). In an attempt to look at the methylation pattern for each insertion separately, we tried mapping the bisulfite reads to the corresponding genome, but the coverage was too low and noisy to observe a difference in methylation between the multiple insertions (Additional file 1: Fig. S14).

Having located precise insertions in the six new genomes, we attempted to find them using short-read data in the 1001 Genomes dataset. Except for one insertion that was shared by 60% of accessions, the rest were found in less than 20%, suggesting that this new element has no fixed insertions in the genome — including the insertion found in the TAIR10 reference genome, which was only found in 17.4% of the accessions (Additional file 1: Fig. S15). We also looked for the element in the genomes of *A. lyrata* (two different genomes), *A. suecica* [a tetraploid containing an *A. thaliana* and an *A. arenosa* subgenome; see 29], and *Capsella rubella* [30]. The gene and the Gypsy element were only found together in *A. thaliana* (including the *A. thaliana* sub-genome of the allopolyploid *A. suecica*). The Gypsy element alone is present in the other *Arabidopsis* species, and the gene alone is present in *A. lyrata*, but only in one of two genomes. In *Capsella rubella*, neither the transposon nor the gene could be detected (Additional file 1: Fig. S16). Thus, the transposon and gene appear to be specific to the genus *Arabidopsis*, while their co-transposition is specific to *A. thaliana*, suggesting that the new transposable element evolved since the divergence of *A. thaliana* from the other member of the genus.

### Spurious methylation polymorphism

Just like cryptic duplications can lead to spurious genetic polymorphisms, they can lead to spurious cytosine methylation polymorphisms. Indeed, given the well-established connection between gene duplication and gene silencing [e.g., [31], they may be more likely to do so. To investigate this, we re-examined the methylation status of genes previously reported by the 1001 Genomes Project [29] as having complex patterns of methylation involving both CG and CHG methylation. In our six sequenced accessions, we found 19530 genes that had been reported as having CG methylation (in at least one accession) and 2556 genes that had been reported as having CHG methylation (in at least one accession). 2473 genes were part of both sets. Out of these, 619, or 24%, had been detected as duplicated in the analyses presented above (a massive enrichment compared to the genome-wide fraction of roughly 10%). To understand these patterns better, we mapped the original bisulfite data to the appropriate genome as well as to the reference genome. In any given accession, roughly 7% of the 2473 genes could not be compared because the homologous copy could not be found (this is presumably mostly because they contain structural variants that prevent them being located by BLAST), and roughly 30% exhibited copy number variation (Table 1). The remaining genes had a single match, almost always in the same location as in the reference genome. These categories are shared across accessions: 1294 of the 2367 genes appeared to be single-copy in all six new genomes, for example (Table 1; Additional files 1-8).

**Table 1:** Number of copies of the 2367 genes identified in each new genome (and Araport11, as control)

Target	Number of copies identified		
	0	1	>1
1254	138	1563	772
5856	174	1566	733
6021	131	1577	765
6024	152	1554	767
9412	147	1567	759
9470	142	1589	742
*Intersection*	*37*	*1294*	*610*
Araport11	0	1721	752

Turning to the methylation patterns, the effect of cryptic copy number variation was obvious (Table 2). For the genes with a single match in both the reference and accession genome, methylation status calls based on mapping bisulfite sequencing reads to either genome were largely concordant (roughly 2.5% disagreement), whereas for genes with copy number variation, roughly one third of calls were wrong.

**Table 2:** Fraction of differentially methylated genes when comparing bisulfite reads mapped to reference TAIR genome and to its respective PacBio genome, separated by gene copy number

Target	Number of copies identified			
	1		>1	
	CG (%)	CHG (%)	CG (%)	CHG (%)
1254	3.0	4.4	33.3	21.6
5856	1.2	3.7	27.8	42.9
6021	2.4	3.2	39.3	24.2
6024	3.0	4.2	41.2	29.5
9412	2.0	2.5	37.0	27.1
9470	2.1	4.7	36.0	26.2

**Fig. 5:**
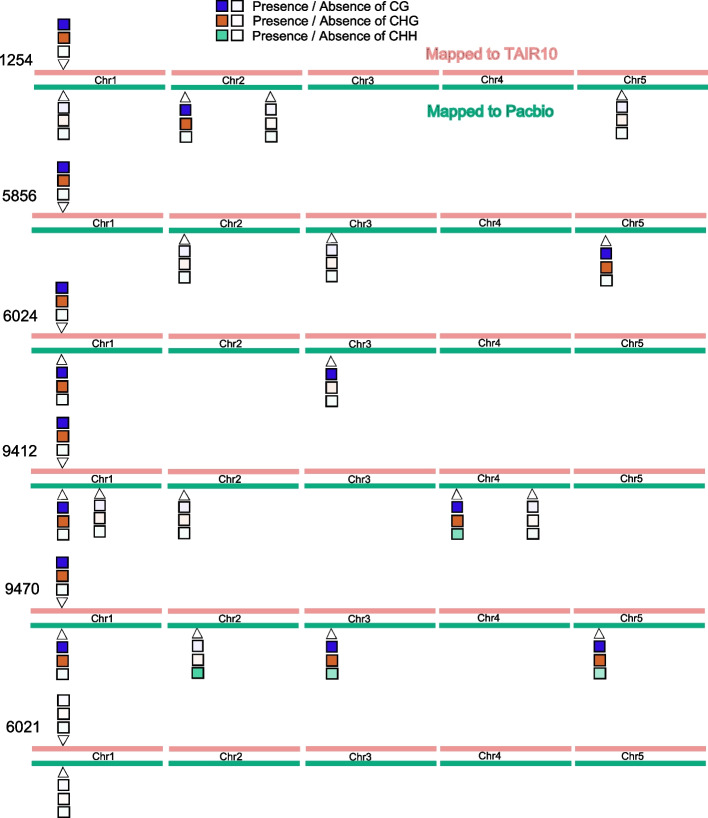
The effect of calling methylation status for AT1G30140 by mapping to a reference genome vs. the appropriate genome. Locations on the chromosomes are approximate, for illustration only

As an illustration for why this occurs, consider the methylation status of AT1G30140 (Fig. 5). When mapped to the reference genome, 5 out of 6 accessions were found to be both CG and CHG methylated, with accession 6021 having no methylation. When mapped to the appropriate genome, we see that this pattern can be quite misleading. In accession 1254, for example, we found three apparent copies of the gene, only two of which are methylated, neither of which is the copy corresponding to the copy present in the reference genome. In accession 5856, the copy corresponding to the reference genome cannot be identified, but a copy on a different chromosome is identified, and it is methylated. In both cases, mapping to the reference genome leads to incorrect methylation status for AT1G30140.

## Discussion

A duplication can lead to pseudo-SNPs when SNPs are identified by mapping short reads to a reference genome that does not contain the duplication. Typically pseudo-SNPs have to be identified using deviations from Hardy-Weinberg proportions, or via non-Mendelian segregation patterns in families or crosses, but in inbred lines, they can be identified solely by their heterozygosity. The overwhelming majority of the 3.3 million heterozygous SNPs identified by our SNP-calling of the 1001 Genomes Project data are likely to be pseudo-SNPs. Assuming this, we used (pseudo-)heterozygosity as a “phenotype,” and tried to map its cause, i.e., the duplication, using a simple but powerful GWAS approach. Focusing on annotated genes, we find that over 2500 (roughly 10% of the total) harbor pseudo-SNPs and show evidence of duplication. Using 6 new long-read assemblies, we were able to confirm 60% of these duplications using conservative criteria (see the “Methods” section). Most of the remaining duplications are located in pericentromeric regions where SNP-calling has lower quality, and which are difficult to assemble even with long-read (Additional file 1: Fig. S17).

These numbers nearly certainly underestimate the true extent of duplication, which has been known to be common in *A. thaliana* for over a decade [32–34]. While unlinked *trans-*duplications are fairly likely to give rise to pseudo-SNPs, local *cis-*duplications will only do so once sufficient time has passed for substantial sequence divergence to occur, or if they arise via non-homologous recombination in a heterozygous individual (which is less likely in *A. thaliana*). As for the GWAS approach, it lacks statistical power to detect rare duplications and can be misled by allelic heterogeneity (due to multiple independent duplications). Finally, duplications are just a subset of structural variants, and it is therefore not surprising that other short-read approaches to detect such variants have identified many more using the 1001 Genomes data [20–22].

Pseudo-SNPs are not the only problem with relying on a reference genome. Our analysis uncovered a striking example of the potential importance of the “mobileome” in shaping genome diversity [35]: we show that an annotated gene and an annotated transposon are both part of a much large mobile element, and the insertion in the reference genome is missing from most other accessions. When short reads from another accession are mapped to this “gene” using the reference genome, you are neither mapping to a gene, nor to the position you think. One possible consequence of this is incorrect methylation polymorphism calls, as we demonstrate above, but essentially any method that relies on mapping sequencing data to a reference genome could be affected (e.g., RNA-seq). It is important that users of such methods be aware of the biases that may result from mapping reads to the “wrong” genome.

Time (and more independently assembled genomes) will tell how significant this problem is, but the potential for artifactual results is clearly substantial, and likely depends on the amount of recent transposon activity [35]. It is also important to realize that the artefactual nature of the 44% heterozygous SNPs was only apparent because we are working with inbred lines. Other researchers working on inbred lines have reached similar conclusions and used various methods to eliminate them, e.g., *Zea* [36–38] and *Brachypodium* [39]. In human genetics, SNP-calling relies heavily on large sample sizes and Hardy-Weinberg assumptions (or even on family trios), but in outcrossing organisms where this is not possible, less precise approaches must be used (e.g., considering the pattern of linkage disequilibrium between putative closely linked SNPs, or variable sequencing coverage). In species with multiple reference genomes, one may be used for quality control, to give an idea of the magnitude of the problem. Ultimately, however, the only real solution to the problem discussed here is moving away from genotyping using short sequence fragments that are prone to mismapping. Our increasing ability to sequence complex genomes will allow population analyses to avoid using such methods and will reveal new mechanisms of genome evolution in the process.

## Conclusions

We have shown that the massive number of heterozygous SNPs identified in the 1001 Arabidopsis Genomes Projects are generally pseudo-heterozygous, mostly due to mis-mapping of reads from segregating duplications, and that many of these duplications can be mapped using GWAS with (pseudo-)heterozygosity as a phenotype. We exemplify the phenomenon by demonstrating that an annotated gene is in fact part of a nearby annotated transposon and that the two generally jumped together, resulting in extensive variation. We note that segregating duplications can bias many analyses and that great caution is needed when mapping short-read sequencing data to the “wrong” genome.

## Methods

### Long-read sequencing of six *A. thaliana*

We sequenced six Swedish *A. thaliana* lines that are part of the 1001 Genomes collection [19], ecotype ids: 1254, 5856, 6021, 6024, 9412, and 9470. Plants were grown in the growth chamber at 21 C in long-day settings for 3 weeks and dark-treated for 24-48 hours before being collected. DNA was extracted from ~20 g of frozen whole seedling material following a high molecular weight DNA extraction protocol adapted for plant tissue [40]. All six genomes were sequenced with PacBio technology, 6021 with PacBio RSII, and the rest with Sequel. The N50 reads length of the accessions 1254, 5856, 6021, 6024, 9412, and 9470 are respectively 27,875, 14,140, 20,989, 28,613, 25,243, and 30,129 bp. Accession 9412 was sequenced twice and 6024 was additionally sequenced with Nanopore (4.1 Gbp sequenced, 376 K reads. All data were used in the assemblies.

### MinION sequencing of two *A. lyrata*

We sequenced two North American *A. lyrata* accessions, 11B02 and 11B21. Both individuals come from the 11B population of *A. lyrata*, which is self-compatible and located in Missouri [41] (GPS coordinates 38° 28′ 07.1″ N; 90° 42′ 34.3″ W). Plants were bulked for 1 generation in the lab and DNA was extracted from ~20g of 3-week-old seedlings, grown at 21°C, and dark treated for 3 days prior to tissue collection. DNA was extracted using a modified protocol for high molecular weight DNA extraction from plant tissue. DNA quality was assessed with a Qubit fluorometer and a Nanodrop analysis. We used a Spot-ON Flow Cell FLO-MIN106D R9 Version with a ligation sequencing kit SQK-LSK109. Bases were called using guppy version 3.2.6 (https://nanoporetech.com/community). The final output of MinION sequencing for 11B02 was 13.67 Gbp in 763,800 reads and an N50 of 31.15 Kb. The final output of MinION sequencing for 11B21 was 17.55 Gb, 1.11 M reads with an N50 of 33.26 Kb.

### Genome assembly, polishing, and scaffolding

The six *A. thaliana* genomes (ecotype ids 1254, 5856, 6021, 6024, 9412, and 9470) were assembled using Canu (v 1.7.1) [42] with default settings, except for genome size. Previous estimates of flow cytometry were used for this parameter [43] when available or 170m was used. The values were 170m, 178m, 135m, 170m, 170m and 170m, respectively. The assemblies were corrected with two rounds of arrow (PacBio’s SMRT Link software release 5.0.0.6792) and one of Pilon [44]. For arrow, the respective long reads were used and for Pilon, the 1001 Genomes DNA sequencing data, plus PCR-free Illumina 150bp data that was generated for accessions 6024 and 9412; lines 5856, 6021, 9470 had available PCR-free data (250bp reads generated by David Jaffe, Broad Institute). This resulted in 125.6Mb, 124.3Mb, 124.5Mb, 124.7Mb, 127.1 Mb, and 128 Mb assembled bases, respectively; contained in 99, 436, 178, 99, 109, and 124 contigs, respectively. For the accessions 1254, 5856, 6024, 9412, and 9470, the average contigs length is 68,880, 25,852, 85,789, 18,7562, and 85,104 bp, respectively. The polished contigs were ordered and scaffolded with respect to the Col-0 reference genome, using RaGOO [45].

We assembled the genome of the two *A. lyrata* accessions 11B02 and 11B21 using Canu [42] (v 1.8) with default settings and a genome size set to 200 Mb. The genomes of 11B02 and 11B21 were contained in 498 and 265 contigs, respectively. The contig assemblies were polished using Racon [46] (v 1.4) and ONT long reads were mapped using nglmr [47] (v 0.2.7). Assemblies were further polished by mapping PCR-free Illumina 150bp short reads (~100X for 11B02 and ~88X for 11B21) to the long-read corrected assemblies. Short-read correction of assembly errors was carried out using Pilon [44] (v1.23). Contigs were scaffolded into pseudo-chromosomes using RaGOO (Alonge et al. 2019) and by using the error-corrected long reads from Canu and the *A. lyrata* reference genome [48] and the *A. arenosa* subgenome of *A. suecica* [49] as a guide followed by manual inspection of regions. The assembly size for 11B02 was 213Mb and 11B21 was 202Mb. Genome size was estimated using findGSE [50] with a resulting estimated genome size of ~256Mb for 11B02 and ~237Mb for 11B21.

### SNPs calling / extraction

We downloaded short-read data for 1,057 accessions from the 1001 Genomes Project [19]. Raw paired-end reads were processed with cutadapt (v1.9) [51] to remove 3′ adapters, and to trim 5′-ends with quality 15 and 3′-ends with quality 10 or N-endings. All reads were aligned to the *A. thaliana* TAIR10 reference genome [52] with BWA-MEM (v0.7.8) [53], and both Samtools (v0.1.18) and Sambamba (v0.6.3) were used for various file format conversions, sorting and indexing [54, 55], while duplicated reads where by marked by Markduplicates from Picard (v1.101; http://broadinstitute.github.io/picard/). Further steps were carried out with GATK (v3.4) functions [26, 56]. Local realignment around indels were done with “RealignerTargetCreator” and “IndelRealigner,” and base recalibration with “BaseRecalibrator” by providing known indels and SNPS from The 1001 Genomes Consortium [19]. Genetic variants were called with “HaplotypeCaller” in individual samples followed by joint genotyping of a single cohort with “GenotypeGVCFs.” An initial SNP filtering was done following the variant quality score recalibration (VQSR) protocol. Briefly, a subset of ~181,000 high-quality SNPs from the RegMap panel [57] was used as the training set for VariantRecalibrator with a priori probability of 15 and four maximum Gaussian distributions. Finally, only bi-allelic SNPs within a sensitivity tranche level of 99.5 were kept, for a total of 7,311,237 SNPs.

### Heterozygous stretches analysis

From the VCF, Plink was used to generate .ped and .map files. (http://pngu.mgh.harvard.edu/purcell/plink/) [58]. To detect and characterize the stretches of heterozygosity the package “detectRUNS” in R was then used. (https://github.com/bioinformatics-ptp/detectRUNS/tree/master/detectRUNS). We used the function slidingRuns.run with the following parameters: WindowSize=10, threshold=0.05, RoHet=True, minDensity=1/100, rest as default.

### SNP filtering

From the raw VCF files SNP positions containing heterozygous labels were extracted using GATK VariantFiltration. From the 3.3 million of heterozygous SNPs extracted, two filtering steps were then applied. Only SNPs with a frequency of at least 5% of the population and located in TAIR10-annotated coding regions were kept. After those filtering steps a core set of 26,647 SNPs were retained for further analysis (see Additional file 1: Fig. S17). Gene names and features containing those pseudo-SNPs were extracted from the TAIR10 annotation.

### GWAS

The presence and absence of pseudo-heterozygosity at a given site (coded as 1 and 0 respectively) was used as a phenotype to run GWAS. As a genotype, the matrix published by the 1001 Genomes Consortium containing 10 million SNPs was used [19]. To run all the GWAS, the pygwas package [https://github.com/timeu/PyGWAS; see [59]] with the amm (accelerated mixed model) option was used. The raw output containing all SNPs was filtered, removing all SNPs with a minor allele frequency below 0.05 and/or a -log10(p-value) below 4.

For each GWAS performed, the *p*-value as well as the position was used to call the peaks using the Fourier transform function in R (filterFFT), combined with the peak detection function (peakDetection), from the package NucleR 3.13, to automatically retrieve the position of each peak across the genome. From each peak, the highest SNPs within a region of +/− 10kb around the peak center were used (see the example in Additional file 1: Fig. S18). Using all 26647 SNPs, a summary table was generated with each pseudo-heterozygous SNP and each GWAS peak detected (Additional file 2). This matrix was then used to generate Fig. 2C, applying thresholds of −log10(*p*-value) of 20 and a minor allele frequency of 0.1.

### Confirmation of GWAS results

To confirm the detected duplications, a combination of BLAST and synteny was used on the denovo-assembled genome. Only the insertions that segregate in the 6 new genomes were used (398). For each gene, the corresponding sequence from the TAIR10 annotation was located in the target genome using BLAST (see Additional file 1: Fig. S6). A threshold of 70% sequence identity as well as 70% of the initial sequence length was used. The presence of a match within 20kb of the predicted peak position was interpreted as confirmation.

### Gene ontology

Out of the 2570 genes detected to be duplicated, 2396 have a gene ontology annotation. PLAZA.4 [60] was used to perform a gene enrichment analysis using the full genome as background. Data were then retrieved and plotted using R.

### Coverage and methylation analysis

Bisulfite reads for the accessions were taken from 1001 methylomes (Kawakatsu et al. 2016). Reads were mapped to PacBio genomes using an nf-core pipeline (https://github.com/rbpisupati/methylseq). We filtered for cytosines with a minimum depth of 3. They methylation levels were calculated either on the gene-body or on 200bp windows using custom python scripts following guidelines from Schultz et al. [61]. Weighted methylation levels were used, i.e., if there are three cytosines with a depth of t1, t2, and t3 and number of methylated reads are c1, c2, and c3, the methylation level was calculated as (c1+c2+c3)/(t1+t2+t3). We called a gene “differentially methylated” if the difference in weighted methylation level was more than 0.05 for CG and 0.03 for CHG.

The sequencing coverage for each accession was extracted using the function bamCoverage (windows size of 50bp) from the program DeepTools [62]. The Bigwig files generated were then processed in R using the package rtracklayer. No correlation between the mean sequencing coverage and the number of pseudo-SNPs detected was observed (Additional file 1: Fig. S18).

### Multiple sequence alignment

For each insertion of the AT1G20390–AT1G20400 (Transposon+gene) fragment, a fasta file including 2kb on each side of the fragment was extracted from each genome, using the getfasta function from bedtools [63]. Multiple alignment was performed using KALIGN [64]. Visualization and comparison were done using Jalview 2 [65].

### Structural variation analysis

To control the structure of the region around duplicated genes, the sequence from 3 genes upstream and downstream of the gene of interest was extracted. Each sequence was then BLAST to each of the genomes and the position of each BLAST result was retrieved. NCBI BLAST [66] was used with a percentage of identity threshold of 70% and all other parameters as default. From each blast results fragments with at least 50% of the input sequence length have been selected and plotted using R.

### Frequency of the insertions in the 1001 Genomes dataset

The same sequences used for the multiple alignment were used to confirm the presence or absence of each insertion in the 1001 Genomes dataset. We used each of those sequences as a reference to map short reads using minimap 2 [67]. For each insertion, only paired-end reads having both members of the pair mapping to the region were retained. An insertion was considered present in an accession if at least 3 pairs of reads spanned the insertion border (see Additional file 1: Fig. S12).

### Multiple species comparison

We used the *Capsella rubella* and *A.arenosa* genomes [30, 49] to search for the new Transposon+gene element, just like in the *A. thaliana* genomes. For *A. arenosa* we used the subgenome of *A. suecica*. We located the transposon+gene fragments, extracted from the TAIR10 annotation, using NCBI BLAST as above. For *A.lyrata* two newly assembled genomes were assembled using MinION sequencing.

## Supplementary Information

**Additional file 1: Fig S1.** Distribution of heterozygosity across individuals and the genome. **Fig S2.** Tract-length distribution. **Fig S3.** SNP filtering scheme. The SNPs matrix we started with contains 7 million SNPs. Of those, 3.3 million were detected as heterozygous in at least one line. We selected the 48,799 SNPs that we called heterozygous in at least 5 % of the lines, and focused on the 26,647 found in coding regions (according to the TAIR10 annotation). **Fig S4.** The distribution of distances between the “position” of a pseudo-SNPs and the corresponding GWAS peak. Out of the 411688 peaks we found that 236494 (57%) are on a different chromosome. The plots show the distribution of distances for the 175194 peaks that were on the same chromosome as the corresponding pseudo-SNPs. Roughly 50% of there are more distance than 50kb. **Fig S5.** Gene ontology analysis of the putatively duplicated genes. **Fig S6.** Pipeline to confirm GWAS peaks. **Fig S7.** Mapping reads tagging singleton pseudo-SNP. First, all reads overlapping the position of a specific pseudo-SNP were extracted based on mapping to the reference genome (TAIR10). This set of reads were then re-mapped to the appropriate Pacbio genome. Reads mapping to multiple regions indicate the presence of a duplicated segment. A decrease in coverage compared to the mapping to the reference genome is also a confirmation that reads map at different positions. An example is presented in **Fig S9**. **Fig S8.** Comparison of mapping coverage between reference genome and PacBio genomes for all regions surrounding pseudo-heterozygous doubleton positions. **Fig S9.** Mapping of reads overlapping singletons (example). **Fig S10.** Position of AT1G31390 in the reference (accession 6909) and each of the six newly assembled genomes. BLAST-thresholds of 70% identity were used, and only fragments of length greater than 50% of the original gene length are shown. **Fig S11.** Position of AT1G20400 in the reference (accession 6909) and each of the six newly assembled genomes. Cf. **Fig S10**. **Fig S12.** Dot-plots of the end of insertion B. LTR repeats can be detected on each side of the insertion. Cf. **Fig S13**. **Fig S13.** Dot plot of the ends of all insertions. Cf. **Fig S12**. **Fig S14.** Methylation profile across all copies of the TE+gene insertions. Bisulfite reads were mapped to the appropriate genomes and profiles extracted based on the inferred locations. Colors distinguish the different insertions found in each genome. **Fig S15.** Frequency of insertions of the new element in the 1001 Genomes. **Fig S16.** Mapping of the AT1G20400 region in multiple species. The rectangle corresponds to annotated genes around AT1G20400 in the A. thaliana reference genome. **Fig S17.** Chromosomal position of putatively duplicated genes that could not be confirmed using full genome sequence. The figure shows the distribution of these genes compared to the distribution of all genes, and RepeatMasker annotation. **Fig S18.** Illustration of GWAS peak calling. The p-values from GWAS were used to run the filterFFT function from the NucleR package in R, using 0.05 for pcKeepComp option. **Fig S19.** The relationship between the number of pseudo-SNPs and sequencing coverage. Each dot represents an accession.

**Additional file 2. **Methylation value per gene of all accessions mapped to the reference genome. CG and CHG weighted average per genes of the 6 accessions analyzed. Row names correspond to the gene ID and column name to the CG and CHG for each accession.

**Additional file 3. **Methylation value per gene of all accessions mapped to the corresponding genome. CG and CHG weighted average per genes of the 6 accessions analyzed. Row names correspond to the gene ID. (the “_” corresponds to the multiple copies detected). The column name to the CG and CHG for each accession.

**Additional file 4. **Methylation value per gene of all accessions mapped to the corresponding genome. CG and CHG weighted average per genes of the 6 accessions analyzed. Row names correspond to the gene ID. (the “_” corresponds to the multiple copies detected). The column name to the CG and CHG for each accession.

**Additional file 5. **Methylation value per gene of all accessions mapped to the corresponding genome. CG and CHG weighted average per genes of the 6 accessions analyzed. Row names correspond to the gene ID. (the “_” corresponds to the multiple copies detected). The column name to the CG and CHG for each accession.

**Additional file 6. **Methylation value per gene of all accessions mapped to the corresponding genome. CG and CHG weighted average per genes of the 6 accessions analyzed. Row names correspond to the gene ID. (the “_” corresponds to the multiple copies detected). The column name to the CG and CHG for each accession.

**Additional file 7. **Methylation value per gene of all accessions mapped to the corresponding genome. CG and CHG weighted average per genes of the 6 accessions analyzed. Row names correspond to the gene ID. (the “_” corresponds to the multiple copies detected). The column name to the CG and CHG for each accession.

**Additional file 8. **Methylation value per gene of all accessions mapped to the corresponding genome. CG and CHG weighted average per genes of the 6 accessions analyzed. Row names correspond to the gene ID. (the “_” corresponds to the multiple copies detected). The column name to the CG and CHG for each accession.

**Additional file 9. **Methylation value per gene of all accessions mapped to the corresponding genome. CG and CHG weighted average per genes of the 6 accessions analyzed. Row names correspond to the gene ID. (the “_” corresponds to the multiple copies detected). The column name to the CG and CHG for each accession.

**Additional file 10. **Review history.

## Data Availability

All genome assemblies and raw reads were deposited under the BioProject ID: PRJNA779205 https://www.ncbi.nlm.nih.gov/bioproject/PRJNA779205 [68]. Scripts used are available under Github with a GPL-3.0 license link: https://github.com/benjj212/duplication-paper.git [69] as well deposited 10.5281/zenodo.7555970 [70]. All scripts are publicly available. The full GWAS matrix is available at 10.5281/zenodo.5702395 [71].
