# Supplementary material for: Extensive sequence duplication in Arabidopsis revealed by pseudo-heterozygosity
Source: Genome Biol. 2023 Mar 9;24:44. doi: 10.1186/s13059-023-02875-3 (PMC9999624; doi:10.1186/s13059-023-02875-3)
Supplement: Supplementary file 10 — Additional file 10. Review history. [file 13059_2023_2875_MOESM10_ESM.docx]

Review History

**First round of review**

**Reviewer 1**

**Were you able to assess all statistics in the manuscript, including the appropriateness of statistical tests used?**

Yes, and I have assessed the statistics in my report.

**Comments to author:**

In their study, Jaegle et al. investigate to what extent cryptic structural variation may lead to spurious heterozygote SNPs. Treating Heterozygosity as a phenotype, the authors used GWAs to map the position of duplicated genes and could therefore untangle cis- from trans- duplications. They further confirm those with Pacbio assemblies.

The analyses are sound but I find the study descriptive. I usually appreciate short formats but as described below, I miss the novelty of the current study, possibly because of the concise introduction/discussion. I also think the manuscript could be improved by citing additional relevant works to place the study in a larger context. For instance,

- L7: "it is becoming apparent". As stated in the introduction chromosome-level assemblies and pangenome studies have revealed that structural variations are ubiquitous of plant genomes. It is therefore not "becoming" but "is" apparent and a problem for trait mapping. Additional works that could be included for instance:

Jayakodi M, Padmarasu S, Haberer G, Bonthala VS, Gundlach H, Monat C, Lux T, Kamal N, Lang D, Himmelbach A, et al.: The barley pan-genome reveals the hidden legacy of mutation breeding. Nature 2020, 588:284-289.

Haberer G, Kamal N, Bauer E, Gundlach H, Fischer I, Seidel MA, Spannagl M, Marcon C, Ruban A, Urbany C, et al.: European maize genomes highlight intraspecies variation in repeat and gene content. Nat Genet 2020, 52:950-957.

Walkowiak S, Gao L, Monat C, Haberer G, Kassa MT, Brinton J, Ramirez-Gonzalez RH, Kolodziej MC, Delorean E, Thambugala D, et al.: Multiple wheat genomes reveal global variation in modern breeding. Nature 2020, 588:277-283.

Wang, W., Mauleon, R., Hu, Z. et al. Genomic variation in 3,010 diverse accessions of Asian cultivated rice. Nature 557, 43-49 (2018).

Major comments:

- L57: "Several groups have used these data to identify large numbers of structural variants". Besides stating that their approach is "broadly applicable" (but see comment below about script availability), and "can reveal interesting biology", the authors do not explain why their study is novel, apart from mapping cis- vs- trans duplication. There is no recommendation on how to treat heterozygosity and no analysis showing to which extent it may bias some (but not all) downstream analyses. This is largely testable with all the data available for Arabidopsis and should at least be developed in the discussion.

- The results section starts abruptly. A little summary about how many heterozygous sites are observed across the 1001 genomes could be useful. Here I also don't understand why TEs are not filtered out, OR at least described separately from genes. TEs are of course know to artificially inflate the number of SNPs and are in many studies filtered out. What part of the 44% is explained by TEs and therefore by already known structural variants? The author state in the discussion that "artifactual results […] likely depends on the amount of recent transposon activity", which seems to be pushing on an open door.

- "broadly applicable": none of the scripts have been archived on the github page mentioned in the main text (reviewed 15th Dec) and assessing more technical aspects of the study is therefore impossible. L378, the author also referred to a "custom python scripts (Schultz et al. 2012) " but the reference is missing. While the authors are known for the quality of their work, this is nonetheless improper in the context of reproducible science.

Minor comments:

Text in figure mostly too small to be read properly.

L414-418: this is part of the result section.

**Reviewer 2**

**Were you able to assess all statistics in the manuscript, including the appropriateness of statistical tests used?**

Yes, and I have assessed the statistics in my report.

**Comments to author:**

This study started with the finding that approximately half of identified SNPs are heterozygous in at least one accession using the raw reads from the 1001 Arabidopsis Genomes Project. As stated by the authors, this incredibly high proportion of heterozygous SNPs were not possible in a highly selfing organism, Arabidopsis. Terming those spurious heterozygous SNPs as pseudo-SNPs, the authors suspected that they may be derived from copy number variants (CNVs) and further validated this using GWAS and six de novo assembled accessions. Finally, some special gene duplication examples were given to illustrate the origin, characteristic and application of identified pseudo-SNPs.

Major:

1. the significance of this paper is limited. It is only found that the missing copies of Arabidopsis reference genome (caused by variety differences or assembly errors) will cause the emergence of pseudo heterozygous SNPs in downstream analysis. Further biological significance needs to be explored.

2. As the pseudo-SNPs were from non-identical duplicates that are missing from the reference genome using mis-mapping sequence reads, it is probably important to evaluate the false positive rate generated by the read alignment tools. In the "Rare duplicates" section, authors mentioned that 40% pseudo-heterozygous SNPs were presented only in one accession, therefore, it is not clear whether these SNPs were from non-reference genome duplicates or alignment errors. A benchmark should increase the reliability of conclusion. It is necessary to provide evidence of duplication of relevant genes based on CNVs identification tools.

3. The authors need to use more direct evidence to show that the pseudo heterozygous SNPs are caused by the CNVs of the genes. For example, the Illumina reads of a certain variety were compared to Pacbio de novo genome and reference genome at the same time, and the changes in the number of heterozygous SNPs on genes with duplication were predicted before statistics.

4. What is the effect of GWAS in this paper? It looks like to screen out which regions (genes) had heterozygous sites in a large number of varieties. Can similar peaks be obtained by directly calculating the distribution of conserved heterozygous SNP loci in 1001 varieties?

5. As indicated in Figure 1B, the distribution of heterozygous SNPs is somewhat accordant with that of TEs. Reads aligner had low sensitivity to map reads in repetitive regions, thus the proportion of gene duplication events may be overestimated by using the methods presented here.

6. What are the three genes in Figure 2? These genes should be detailed analyzed.

**Reviewer 3**

**Were you able to assess all statistics in the manuscript, including the appropriateness of statistical tests used?**

No, I do not feel adequately qualified to assess the statistics.

**Comments to author:**

I find the manuscript original and worth being published in Genome Biology, even though the applicability of the method utilised to identify CNVs is limited to selfing species where highly inbred individuals are available for the resequencing.

The manuscript requires quite extensive language editing because many sentences are not very clear. There are a number of places where the poor writing of either the text or the figure captions makes it quite difficult to follow the presentation of the results. As an example the reference genome is sometime indicated as TAIR10, sometime as 6909 (at least so I think).

I think the manuscript would greatly benefit from a better exploitation of the information obtained from the 6 newly assembled genomes. While it is certainly interesting to learn that a significant number of genes are present in a variable number of copies across the 1001 genomes, it would be even more interesting to learn more about the mechanisms driving the duplications, especially the so called trans duplications that form the vast majority of those identified through the GWAS approach utilised here. So I would strongly encourage the authors to perform a few additional analyses even if only on a subset of all genes validated in the 6 assembled accessions in order to gain the following information:

1) what fraction of events is related to TE movement and transduplication events carried out by elements such as Helitrons or PackMules or CACTA TEs or any other TE

2) how large is the duplicated region: is it limited to the transcribed portion or to this plus 5' and 3' flanking regions etc.

3) is the duplication mechanism related to unequal homologous recombination or to microhomology-mediated end joining or some other identifiable mechanism?

Another concern I have is related to the discussion on the AT1G20400 gene duplication. While I find convincing the evidences provided to support the hypothesis that the mechanism that led to the duplication is related to the movement of a transposable element, I find much less convincing the evidences provided that AT1G20400 is a real gene. Functional evidences for transcription are not provided nor for presence of a phenotype in mutants for the gene. It would be important to provide additional information that supports the claim that it is indeed a gene.

**Authors Response**

**Point-by-point responses to the reviewers’ comments:**

Reviewer #1

In their study, Jaegle et al. investigate to what extent cryptic structural variation may lead to spurious heterozygote SNPs. Treating Heterozygosity as a phenotype, the authors used GWAs to map the position of duplicated genes and could therefore untangle cis- from trans-duplications. They further confirm those with Pacbio assemblies.

The analyses are sound but I find the study descriptive. I usually appreciate short formats but as described below, I miss the novelty of the current study, possibly because of the concise introduction/discussion. I also think the manuscript could be improved by citing additional relevant works to place the study in a larger context. For instance,

- L7: "it is becoming apparent". As stated in the introduction chromosome-level assemblies and pangenome studies have revealed that structural variations are ubiquitous of plant genomes. It is therefore not "becoming" but "is" apparent and a problem for trait mapping. Additional works that could be included for instance:

Jayakodi M, Padmarasu S, Haberer G, Bonthala VS, Gundlach H, Monat C, Lux T, Kamal N, Lang D, Himmelbach A, et al.: The barley pan-genome reveals the hidden legacy of mutation breeding. Nature 2020, 588:284-289.

Haberer G, Kamal N, Bauer E, Gundlach H, Fischer I, Seidel MA, Spannagl M, Marcon C, Ruban A, Urbany C, et al.: European maize genomes highlight intraspecies variation in repeat and gene content. Nat Genet 2020, 52:950-957.

Walkowiak S, Gao L, Monat C, Haberer G, Kassa MT, Brinton J, Ramirez-Gonzalez RH, Kolodziej MC, Delorean E, Thambugala D, et al.: Multiple wheat genomes reveal global variation in modern breeding. Nature 2020, 588:277-283.

Wang, W., Mauleon, R., Hu, Z. et al. Genomic variation in 3,010 diverse accessions of Asian cultivated rice. Nature 557, 43-49 (2018).

*Reply: We agree that the introduction was too concise, and failed to emphasize that the observation of structural variation is by no means novel. We have remedied this, and also tried to clarify the point of our paper, which is that, even in A. thaliana, a selfing plant with a tiny and relatively simple genome, and with what is almost certainly the best annotation, there are massive problems. We further point to some very simply methods that can be used to gain insight into the magnitude of the problem - at least in selfers (which includes many major crops). In the revision, we have also added an analysis demonstration that cryptic structural variation impacts other analyses (see below).*

Major comments:

- L57: "Several groups have used these data to identify large numbers of structural variants". Besides stating that their approach is "broadly applicable" (but see comment below about script availability), and "can reveal interesting biology", the authors do not explain why their study is novel, apart from mapping cis- vs- trans duplication. There is no recommendation on how to treat heterozygosity and no analysis showing to which extent it may bias some (but not all) downstream analyses. This is largely testable with all the data available for Arabidopsis and should at least be developed in the discussion.

*Reply: Yes, this is certainly the case, and we have taken a step in this direction by including a re-evaluation of the complex genic DNA methylation patterns reported in Kawakatsu et al. (2016). We show that roughly a third of the genes with such patterns exhibit copy-number variation, and that, as a result, roughly a third of reported methylation classifications may be wrong. It goes without saying that this casts doubts on attempts to mine published data for aberrant methylation patterns.*

*A more comprehensive re-evaluation of all techniques and published results that rely on mapping short reads (be it RNA-seq, bs-Seq, or ChIP-seq) is clearly in order, but doing this is a fairly massive undertaking that requires comparing/aligning large numbers of fully sequenced and independently annotated genomes. As part of a consortium, we are currently working on such a study for Arabidopsis, but this is well beyond the scope of this paper.*

*As for how to treat heterozygosity, there is no magic bullet, and we make this clear in the discussion. Ultimately we hope biology moves beyond using reference genomes, but until then, the best we can do is to be aware of the problem, and interpret results with caution. This is the message of the paper.*

- The results section starts abruptly. A little summary about how many heterozygous sites are observed across the 1001 genomes could be useful. Here I also don't understand why TEs are not filtered out, OR at least described separately from genes. TEs are of course know to artificially inflate the number of SNPs and are in many studies filtered out. What part of the 44% is explained by TEs and therefore by already known structural variants? The author state in the discussion that "artifactual results [...] likely depends on the amount of recent transposon activity", which seems to be pushing on an open door.

*Reply: We expanded the start of this section to clarify the SNP calling. Throughout we have tried to emphasize that TEs were of course filtered out - the point is that what is repetitive in the reference genome is only a subset of what is repetitive and copy-number-variable in the population. The answer to the question "What part of the 44% is explained by TEs and therefore by already known structural variants?" is therefore: "Most of it is probably explained by TEs, but not the ones we know of!" We will have the answer when we have sequenced all the genomes.*

- "broadly applicable": none of the scripts have been archived on the github page mentioned in the main text (reviewed 15th Dec) and assessing more technical aspects of the study is therefore impossible. L378, the author also referred to a "custom python scripts (Schultz et al. 2012) " but the reference is missing. While the authors are known for the quality of their work, this is nonetheless improper in the context of reproducible science.

*Reply: We agree, and apologize. The scripts had been uploaded, but were not available. This has been remedied.*

Minor comments:

Text in figure mostly too small to be read properly.

L414-418: this is part of the result section

*Reply: Fixed.*

Reviewer #2

This study started with the finding that approximately half of identified SNPs are heterozygous in at least one accession using the raw reads from the 1001 Arabidopsis Genomes Project. As stated by the authors, this incredibly high proportion of heterozygous SNPs were not possible in a highly selfing organism, Arabidopsis. Terming those spurious heterozygous SNPs as pseudo-SNPs, the authors suspected that they may be derived from copy number variants (CNVs) and further validated this using GWAS and six de novo assembled accessions. Finally, some special gene duplication examples were given to illustrate the origin, characteristic and application of identified pseudo-SNPs.

Major:

**1.** the significance of this paper is limited. It is only found that the missing copies of Arabidopsis reference genome (caused by variety differences or assembly errors) will cause the emergence of pseudo heterozygous SNPs in downstream analysis. Further biological significance needs to be explored.

*Reply: We have added a section demonstrating the cryptic copy number variation likely causes extremely high error rates in assigning methylation states to genes. We have also noted that similar problems likely apply to RNA-seq, ChIP-seq, and any other method that relies on mapping short-reads to the wrong genome.*

**2.** As the pseudo-SNPs were from non-identical duplicates that are missing from the reference genome using mis-mapping sequence reads, it is probably important to evaluate the false positive rate generated by the read alignment tools. In the "Rare duplicates" section, authors mentioned that 40% pseudo-heterozygous SNPs were presented only in one accession, therefore, it is not clear whether these SNPs were from non-reference genome duplicates or alignment errors. A benchmark should increase the reliability of conclusion. It is necessary to provide evidence of duplication of relevant genes based on CNVs identification tools.

*Reply: The results in the Rare duplicates section have been clarified. They are certainly not from read-alignment errors in the trivial sense of mapping to several places in a known genome. As for CNV identification tools, it is our experience that these are usually noisy, and we go well beyond them by actually using newly assembled genomes and GWAS to confirm the location of duplicated genes.*

**3.** The authors need to use more direct evidence to show that the pseudo heterozygous SNPs are caused by the CNVs of the genes. For example, the Illumina reads of a certain variety were compared to Pacbio de novo genome and reference genome at the same time, and the changes in the number of heterozygous SNPs on genes with duplication were predicted before statistics.

*Reply: Good point - they do indeed largely disappear, and we now mention this.*

**4.** What is the effect of GWAS in this paper? It looks like to screen out which regions (genes) had heterozygous sites in a large number of varieties. Can similar peaks be obtained by directly calculating the distribution of conserved heterozygous SNP loci in 1001 varieties?

*Reply: No, the point of GWAS is to map the location of the cryptic duplication, which, by definition, is not present in the reference genome. The distribution of heterozygous SNPs just reveals where genes and regions affected by this are located.*

**5.** As indicated in Figure 1B, the distribution of heterozygous SNPs is somewhat accordant with that of TEs. Reads aligner had low sensitivity to map reads in repetitive regions, thus the proportion of gene duplication events may be overestimated by using the methods presented here.

*Reply: SNP calling does take known repeats into account, but cannot do anything about unknown (cryptic) ones. This is the whole point of the paper.*

**6.** What are the three genes in Figure 2? These genes should be detailed analyzed.

*Reply: They are just random examples, for illustration. This is now explicitly stated in the caption.*

Reviewer #3

I find the manuscript original and worth being published in Genome Biology, even though the applicability of the method utilised to identify CNVs is limited to selfing species where highly inbred individuals are available for the resequencing.

The manuscript requires quite extensive language editing because many sentences are not very clear. There are a number of places where the poor writing of either the text or the figure captions makes it quite difficult to follow the presentation of the results. As an example the reference genome is sometime indicated as TAIR10, sometime as 6909 (at least so I think).

*Reply: We agree about the language, and have attempted to remedy this throughout. Apologies! We now consistently use TAIR10 to refer to the reference genome.*

I think the manuscript would greatly benefit from a better exploitation of the information obtained from the 6 newly assembled genomes. While it is certainly interesting to learn that a significant number of genes are present in a variable number of copies across the 1001 genomes, it would be even more interesting to learn more about the mechanisms driving the duplications, especially the so called trans duplications that form the vast majority of those identified through the GWAS approach utilised here. So I would strongly encourage the authors to perform a few additional analyses even if only on a subset of all genes validated in the 6 assembled accessions in order to gain the following information:

**1)** what fraction of events is related to TE movement and transduplication events carried out by elements such as Helitrons or PackMules or CACTA TEs or any other TE

**2)** how large is the duplicated region: is it limited to the transcribed portion or to this plus 5' and 3' flanking regions etc.

**3)** is the duplication mechanism related to unequal homologous recombination or to microhomology-mediated end joining or some other identifiable mechanism?

*Reply: We absolutely agree with all these points, but they are far beyond the scope of this paper. We are working on a very large paper in collaboration with Detlef Weigel and Paul Kersey in which 27 independently annotated genomes (with transcriptomes from multiple tissues) will be compared, partly to gain insight into the nature of the mechanisms. We know from years of experience that these are not trivial questions to answer if you want to get it right. We have added a relatively simple analysis of the length distribution of the duplicated regions that suggested that a large fraction of them are fragments (Supplemental Figure 5). The fact that many also show transposon-methylation strongly suggests the involvement of transposons, but going beyond that requires a lot of multiple-sequence alignment as well as method development.*

Another concern I have is related to the discussion on the AT1G20400 gene duplication. While I find convincing the evidences provided to support the hypothesis that the mechanism that led to the duplication is related to the movement of a transposable element, I find much less convincing the evidences provided that AT1G20400 is a real gene. Functional evidences for transcription are not provided nor for presence of a phenotype in mutants for the gene. It would be important to provide additional information that supports the claim that it is indeed a gene.

*Reply: In the revised version we make clear that we only think of AT1G20400 as an annotated gene, and we do not actually make any statement about it being functional. In fact, we are convinced that many of the "genes" that show CNV are not actually genes, but rather part of the "mobileome". In a sense, the main message is that: a) the TAIR10 annotation does not necessarily transfer to other lines, and; b) the TAIR10 annotation is not even to be trusted for Col-0.*

**Second round of review**

**Reviewer 2**

Although the authors have revised the manuscript, and addressed most of my comments, I still think the novelty of this study is not enough to be published in Genome Biology. The analysis is too descriptive and lack of enough biological significance.

**Reviewer 4**

The article brings novel and very original view on the 1001 genome data. The findings that large proportion of SNPs are in fact artifact of structural variation (SV) is very informative and valuable for any thaliana-diversity studying researcher. Validation using PacBio long read data is very valuable addition and informs about the power and limits of the short-read approach. I thus consider this paper very innovative and interesting for broad spectrum of readers. I have just couple of minor issues

1) l 101-103 Esp. when reading the Results section it is unclear how the "pseudo heterozygous genes" were defined. The "5% segregation" threshold means the site was heterozygous in at least ~50 individuals out of the 1001? (or or even more given the dataset contains 1135 accessions?) Yet the text before speaks only about 5 individuals (l 84-85). And how such gene was defined - containing at elast one such >5% het. SNP or minimum of N heterozygous SNPs (a "tract")?

2) I wonder how much neutral divergence can affect the presence/absence of gene duplications /deletions and thus the proportion of pseudo-heterozygosity. How was the variation in % of het SNPs out of teh total across accessions? As there is quite a strong population structure in the 1001 dataset (e.g. relict lineages) I whonder whether neutral divergence from the reference inferred from "rela SNPs" may correlate with % of SNPs identified as pseudo-heterozygotes. Such information may provide useful hints for both evolutionary history signal in SVs and for practical considerations.

Minors

l. 24 - "most het SNPs are artifacts" - although this makes sense, for saying "most" a quantification seems to be lacking in the paper

l. 102 - it is what is a "population" - is it a complete dataset of 1135 genomes or just a subset of closely related lineages?

l. 82 "...the same SNP IS..."

**Reviewer 5**

In this manuscript, Jaegle et al. begin their investigation from the observation that Arabidopsis thaliana genetic variation contain a high frequency of heterozygous SNPs. As this species is mostly a selfer, this observation is suspicious, and the authors suggest that most of the observed heterozygosity is in fact due to "pseudo-SNPs" derived from pervasive gene copy-number-variation (CNV) across A. thaliana ecotypes. An innovative use of GWAS is applied to detect the genomic position of gene duplicates, and the authors further show that cryptic CNVs affect the analysis of DNA methylation patterns. This work points at an important methodological bias as well as an interesting biological phenomenon. The manuscript is mostly well written, however, the description of the GWAS methodology and its connection to the duplication placements is not sufficient. However, we have some concerns regarding the reliability of the results due to some methodological uncertainties. We believe that some additional analyses are still required to make this work more complete and convincing. Our comments and suggestions to the authors are listed below.

Major comments

1. We believe it should be relatively easy to provide additional support that the inferred pseudo-SNPs are derived from gene duplications. A more direct line of evidence that the observed heterozygosity derives from gene duplication would be by looking at sequencing depth. More specifically, by showing that the sequencing depth in regions where heterozygous SNPs occur is higher than other regions (using some statistical test).

2. Similar to point 1 above, the shared heterozygote SNPs are presumably due to an ancestral duplication that gave rise to the same duplicated region in multiple individuals. The authors can thus show this for the over million heterozygous SNPs are shared by at least 5 accessions. In these - we expect to see a strong phylogenetic signal.

3. The authors make use of non-reference genomes from the Jiao 2019 paper as well as those de novo assembled in this study. It would be interesting to use these full genome sequences to reconstruct the various haplotypes of gene copies found within a genome and among the population. This way we can determine how different they are from one another and e.g. estimate divergence times, reconstruct the evolution of gene duplication and determine if a phylogenetic signal is present. More importantly, we could determine how similar two gene copies need to be to produce pseudo-SNPs.

Other comments

1. Line 33: "translocations" would be more general than "transpositions".

2. Line 41: missing comma after "paired-end mapping"

3. Line 41: "depth" or "depth of coverage" would be a more appropriate term than "coverage".

4. Line 66: The sentence beginning with "Note that allelic SNP differences…" is unclear. Are you referring to cases where outcrossing occurred? If so, please explain it explicitly.

5. Line 91: Is there a way to assess the expected length of heterozygous blocks under some null model, and see how different the observed length distribution is from that expectation? Performing a statistical test could provide a significance measure.

6. Line 101: what are the "evidence for selection"? can you be more explicit?

7. Lines 107-109: even after looking at the figure and reading the relevant Methods section, it is still not entirely clear how the GWAS approach works. This description must be expanded, allowing readers clearer understanding of the analysis and how it was used to detect the position of gene duplicates. The authors try to illustrate this in figure 2, but it is still not clear from this figure how GWAS reveals the location of the duplicated region.

8. Line 113: why was 50kb chosen as the cis/trans cutoff? The authors can present the distribution of distances and provide some statistics (same/other chromosome etc.) this would make more sense.

9. Line 117: should provide some sequencing and assembly stats, so readers can assess the quality of genome assemblies (assembly size, N50, BUSCO scores etc.). Some statistics are provided in the Methods section, but others are missing.

10. Line 118: as specified in the Methods, ONT and Illumina sequencing are also used, so "a hybrid approach" would be more accurate than "long read PacBio".

11. Line 159: this entire section is focused on a single example, while containing no genome-wide analysis. It is therefore difficult to tell how common the involvement of TEs in gene duplication is. A more general analysis is required, especially given the title of the section.

12. Lines 181-183. This sentence should be better linked with the previous one to explain the meaning of this analysis.

13. Line 203-204. "…they may be more likely to do so" - needs rephrasing.

14. It would also be interesting to collect some statistics regarding the frequency of gene CNV in the 1001G population - how many genes display such variation and in what fraction of the population? How is the copy number distributed in the population? Such an analysis can be an extension to common pan-genome gene presence-absence analyses.

15. Line 244: do you mean "solely by the presence of heterozygous SNPs"?

16. Line 245: this sentence is a bit confusing. What do "majority" and "44%" refer to?

17. Lines 282-283: Can you expand on that? How is the use of additional assembled genomes expected to improve the situation with regard to the problem you describe?

18. The discussion could benefit from some suggestions on how one can avoid false SNP calling or erroneous methylation detections. What approaches/protocols should be developed or applied?

19. Line 336: the title is misleading, since this section describes SNP calling in general and says nothing about heterozygosity detection.

20. Line 370: maybe adding "…pseudo heterozygosity at a given site…" would make it clearer.

21. Regarding the GWAS analysis - the authors use a python package called pyGWAS. As far as we can tell, this tool had not been published, and the github page does not contain much information. Therefore, some further information should be provided, with the most important point being how the authors control for population structure and for multiple testing. It could also beneficial to repeat the GWAS analysis with one of the many available and popular tools.

**Authors Response**

**Point-by-point responses to the reviewers’ comments:**

**Reviewer #2**

Although the authors have revised the manuscript, and addressed most of my comments, I still think the novelty of this study is not enough to be published in Genome Biology. The analysis is too descriptive and lack of enough biological significance.

**Reviewer 4**

The article brings novel and very original view on the 1001 genome data. The findings that large proportion of SNPs are in fact artifact of structural variation (SV) is very informative and valuable for any thaliana-diversity studying researcher. Validation using PacBio long read data is very valuable addition and informs about the power and limits of the short-read approach. I thus consider this paper very innovative and interesting for broad spectrum of readers. I have just couple of minor issues

1) l 101-103 Esp. when reading the Results section it is unclear how the "pseudo heterozygous genes" were defined. The "5% segregation" threshold means the site was heterozygous in at least ~50 individuals out of the 1001? (or or even more given the dataset contains 1135 accessions?) Yet the text before speaks only about 5 individuals (l 84-85). And how such gene was defined - containing at elast one such >5% het. SNP or minimum of N heterozygous SNPs (a "tract")?

*Reply: This has been clarified in the text.*

2) I wonder how much neutral divergence can affect the presence/absence of gene duplications /deletions and thus the proportion of pseudo-heterozygosity. How was the variation in % of het SNPs out of teh total across accessions? As there is quite a strong population structure in the 1001 dataset (e.g. relict lineages) I whonder whether neutral divergence from the reference inferred from "rela SNPs" may correlate with % of SNPs identified as pseudo-heterozygotes. Such information may provide useful hints for both evolutionary history signal in SVs and for practical considerations.

*Reply: The pseudo-SNPs are generally strongly correlated with other SNPs, and show similar population structure. There are more pseudo-SNPs in more polymorphic regions of the genome, and so on. We don’t say much about this as it is the expectation, and there is nothing terribly interesting to say.*

Minors

l. 24 - "most het SNPs are artifacts" - although this makes sense, for saying "most" a quantification seems to be lacking in the paper.

*Reply: It is obvious from looking at the data that there are few tracts of genuine heterozygosity — as expected given that we have already removed lines that showed evidence of this. We changed the writing (early in Abstract) to emphasize this.*

l. 102 - it is what is a "population" - is it a complete dataset of 1135 genomes or just a subset of closely related lineages?

*Reply: The sentence has been rephrased to clarify.*

l. 82 "...the same SNP IS..."

*Reply: Fixed.*

**Reviewer 5**

In this manuscript, Jaegle et al. begin their investigation from the observation that Arabidopsis thaliana genetic variation contain a high frequency of heterozygous SNPs. As this species is mostly a selfer, this observation is suspicious, and the authors suggest that most of the observed heterozygosity is in fact due to "pseudo-SNPs" derived from pervasive gene copy-number-variation (CNV) across A. thaliana ecotypes. An innovative use of GWAS is applied to detect the genomic position of gene duplicates, and the authors further show that cryptic CNVs affect the analysis of DNA methylation patterns. This work points at an important methodological bias as well as an interesting biological phenomenon. The manuscript is mostly well written, however, the description of the GWAS methodology and its connection to the duplication placements is not sufficient. However, we have some concerns regarding the reliability of the results due to some methodological uncertainties. We believe that some additional analyses are still required to make this work more complete and convincing. Our comments and suggestions to the authors are listed below.

Major comments

1. We believe it should be relatively easy to provide additional support that the inferred pseudo-SNPs are derived from gene duplications. A more direct line of evidence that the observed heterozygosity derives from gene duplication would be by looking at sequencing depth. More specifically, by showing that the sequencing depth in regions where heterozygous SNPs occur is higher than other regions (using some statistical test).

*Reply: As noted in the manuscript, sequencing depth turns out to be quite noisy, and is far less reliable than directly searching for the duplications in long-read assemblies.*

2. Similar to point 1 above, the shared heterozygote SNPs are presumably due to an ancestral duplication that gave rise to the same duplicated region in multiple individuals. The authors can thus show this for the over million heterozygous SNPs are shared by at least 5 accessions. In these - we expect to see a strong phylogenetic signal.

*Reply: Excellent point. We now note that 97% of pseudo-heterozygous SNPs also find both homozygous genotypes — as predicted if these SNPs are ancestral.*

3. The authors make use of non-reference genomes from the Jiao 2019 paper as well as those de novo assembled in this study. It would be interesting to use these full genome sequences to reconstruct the various haplotypes of gene copies found within a genome and among the population. This way we can determine how different they are from one another and e.g. estimate divergence times, reconstruct the evolution of gene duplication and determine if a phylogenetic signal is present. More importantly, we could determine how similar two gene copies need to be to produce pseudo-SNPs.

*Reply: Yes, we are working on a paper together with the Weigel and Kersey groups that does a comprehensive analysis along these lines for 27 genomes.*

Other comments

1. Line 33: "translocations" would be more general than "transpositions".

*Reply: Done.*

2. Line 41: missing comma after "paired-end mapping"

*Reply: Done.*

3. Line 41: "depth" or "depth of coverage" would be a more appropriate term than "coverage".

*Reply: Done.*

4. Line 66: The sentence beginning with "Note that allelic SNP differences…" is unclear. Are you referring to cases where outcrossing occurred? If so, please explain it explicitly.

*Reply: Yes, and done (we omitted this because outcrossing occurred in all cases).*

5. Line 91: Is there a way to assess the expected length of heterozygous blocks under some null model, and see how different the observed length distribution is from that expectation? Performing a statistical test could provide a significance measure.

*Reply: We think this is redundant given the evidence from sharing of heterozygous sites. The pattern illustrated in Figure 1 has probability zero under a model of heterozygosity due to outcrossing.*

6. Line 101: what are the "evidence for selection"? can you be more explicit?

*Reply: Rephrased to clarify.*

7. Lines 107-109: even after looking at the figure and reading the relevant Methods section, it is still not entirely clear how the GWAS approach works. This description must be expanded, allowing readers clearer understanding of the analysis and how it was used to detect the position of gene duplicates. The authors try to illustrate this in figure 2, but it is still not clear from this figure how GWAS reveals the location of the duplicated region.

*Reply: We added some text to clarify, however, ultimately this is one of those conceptual things where some readers will have to draw some figures themselves to understand what is going on.*

8. Line 113: why was 50kb chosen as the cis/trans cutoff? The authors can present the distribution of distances and provide some statistics (same/other chromosome etc.) this would make more sense.

*Reply: 50 kb is roughly the decay of linkage disequilibrium in these data (this has been noted), however, the precise distinction is not important for the argument. The distribution of distances has been added and shows a rapid decay, as expected.*

9. Line 117: should provide some sequencing and assembly stats, so readers can assess the quality of genome assemblies (assembly size, N50, BUSCO scores etc.). Some statistics are provided in the Methods section, but others are missing.

*Reply: This has been added.*

10. Line 118: as specified in the Methods, ONT and Illumina sequencing are also used, so "a hybrid approach" would be more accurate than "long read PacBio".

*Reply: We changed the title of this section accordingly.*

11. Line 159: this entire section is focused on a single example, while containing no genome-wide analysis. It is therefore difficult to tell how common the involvement of TEs in gene duplication is. A more general analysis is required, especially given the title of the section.

*Reply: We agree, however, a more general analysis is beyond the scope of this paper. As noted above, we are working on a comprehensive analysis, and let’s just say it will be ten times the length of this paper. We changed the title instead.*

12. Lines 181-183. This sentence should be better linked with the previous one to explain the meaning of this analysis.

*Reply: Done.*

13. Line 203-204. "…they may be more likely to do so" - needs rephrasing.

*Reply: We don’t see why. Please read again.*

14. It would also be interesting to collect some statistics regarding the frequency of gene CNV in the 1001G population - how many genes display such variation and in what fraction of the population? How is the copy number distributed in the population? Such an analysis can be an extension to common pan-genome gene presence-absence analyses.

*Reply: Again, beyond the scope of this paper. See comments above.*

15. Line 244: do you mean "solely by the presence of heterozygous SNPs"?

*Reply: Yes, fixed.*

16. Line 245: this sentence is a bit confusing. What do "majority" and "44%" refer to?

*Reply: Fixed.*

17. Lines 282-283: Can you expand on that? How is the use of additional assembled genomes expected to improve the situation with regard to the problem you describe?

*Reply: Clarified. No need to “call SNPs” any more.*

18. The discussion could benefit from some suggestions on how one can avoid false SNP calling or erroneous methylation detections. What approaches/protocols should be developed or applied?

*Reply: Well, sequence the genomes, to be blunt. The method of detecting SNPs by aligning reads to a reference genome will soon go the way of the method of detecting SNPs by annealing short fragments to a chip. This is what our final sentence is saying. Until then, be worried, especially if working in an outcrossing organism. We do note that methods like trios work well.*

19. Line 336: the title is misleading, since this section describes SNP calling in general and says nothing about heterozygosity detection.

*Reply: Fixed.*

20. Line 370: maybe adding "…pseudo heterozygosity at a given site…" would make it clearer.

*Reply: Done.*

21. Regarding the GWAS analysis - the authors use a python package called pyGWAS. As far as we can tell, this tool had not been published, and the github page does not contain much information. Therefore, some further information should be provided, with the most important point being how the authors control for population structure and for multiple testing. It could also beneficial to repeat the GWAS analysis with one of the many available and popular tools.

*Reply: It has been published. Reference added.*

**Third round of review**

**Reviewer 6**

In the present manuscript, Jaegle et al. presented the description and investigation of the phenomenon that cryptic CNVs may cause bias of SNP calling using short-read sequencing data. The authors also developed a GWAS method to identify these cryptic CNVs using these pseudo-SNPs as phenotypes. These results are informative, especially for future researchers while they are working on SNPs derived from short-read sequencing data. The GWAS method is also innovative and interesting. However, the manuscript is overall too simple and mainly descriptive, and lack of addressing substantial biological questions. Furthermore, as the title of the manuscript being "Extensive gene duplication in Arabidopsis revealed by pseudo heterozygosity", I would expect more comprehensive analysis on the gene duplication in Arabidopsis, in the genome and population level, which is absent in the manuscript. The authors claimed that these were beyond their scope, so I am confused about the main scope of this study. Only describing the present and cause of technical bias, and developing the GWAS method, without substantially addressing the biological questions are not enough. Therefore I suggest the authors should add more analysis related to the biological meaning of the gene duplication in Arabidopsis.

I also have another major concern about the technical issues in the manuscript. I would suggest the authors could provide some practical approaches to solve the pseudo-SNPs bias while using short-read sequencing data. Actually, we have observed the same phenomenon in our previous study, and we found these pseudo-SNPs will significantly affect the downstream genomic or genetic analysis. For instance, these pseudo-SNPs caused a strong false positive association in our GWAS analysis. Since the authors can identify the causal CNV by their GWAS method, I wonder is that possible to incorporate the CNV GWAS results into the downstream analysis to eliminate this bias. Pan-genome is another possible tool to solve this problem. Since to some extent, pan-genome can collect and precisely present the CNVs, which will reduce the mapping error caused by CNVs. I don't agree with the authors' comment that the only way for researchers to avoid this bias is to be worried, which is meaningless. In my opinion, solving the technical bias based on present tools and technologies is still very valuable for most of the researchers while they use these genomic data.
